# Designing a Tool System for Lowering Friction during the Ejection of In-Die Sintered Micro Gears

**DOI:** 10.3390/mi8070214

**Published:** 2017-07-06

**Authors:** Emanuele Cannella, Emil Krabbe Nielsen, Alessandro Stolfi

**Affiliations:** 1 IPU Product Development, Kgs. Lyngby, 2800 DK, Denmark; 2 DTU Department of Mechanical Engineering, Kgs. Lyngby, 2800 DK, Denmark; alesto@mek.dtu.dk; 3 DTU Department of Electrical Engineering, Kgs. Lyngby, 2800 DK, Denmark; ekrani@elektro.dtu.dk

**Keywords:** micro sintering, field assisted sintering technique, hot isostatic pressing, micro gears, computed tomography, dimensional accuracy, porosity analysis

## Abstract

The continuous improvements in micro-forging technologies generally involve process, material, and tool design. The field assisted sintering technique (FAST) is a process that makes possible the manufacture of near-net-shape components in a closed-die setup. However, the final part quality is affected by the influence of friction during the ejection phase, caused by radial expansion of the compacted and sintered powder. This paper presents the development of a pre-stressed tool system for the manufacture of micro gears made of aluminum. By using the hot isostatic pressing (HIP) sintering process and different combinations of process parameters, the designed tool system was compared to a similar tool system designed without a pre-stressing strategy. The comparison between the two tool systems was based on the ejection force and part fidelity. The ejection force was measured during the tests, while the part fidelity was documented using an optical microscope and computed tomography in order to obtain a multi-scale characterization. The results showed that the use of pre-stress reduced the porosity in the gear by 40% and improved the dimensional fidelity by more than 75% compared to gears produced without pre-stress.

## 1. Introduction

Micro-sintering enables the manufacture of micro-scale components with a complex shape and geometry. Near-net-shape components can be easily manufactured and post-removal processes, e.g., grinding, are limited to the final surface polishing. Compared to cutting processes, since the material scrap is reduced, a high saving of material is possible. Generally, the conventional sintering process can be considered as two different phases, i.e., compaction and heating of the powder [1]. The powder is fed into a closed-die and the punches apply the desired pressure to compact the raw material. The compacted sample, called “green compact”, is ejected from the die and moved into a temperature-controlled oven. The sintering temperature is a process parameter and is strictly dependent on the material. This value never overcomes the melting temperature of the material. Therefore, the green compact is kept inside the oven for a defined time, namely the “sintering time”. In a final phase, the sample is moved out from the oven and cooled down to the room temperature. The whole process makes it possible to enable the densification of the sample by diffusional mechanisms issued from the pressure and high temperature. Since, in the sintering process, the two steps, i.e., heating and compaction, are carried out separately, the manufacturing time per component is extended. Additionally, oxidation of the material may occur and a protective atmosphere is required to prevent this phenomenon.

To decrease the sintering time per component, new sintering processes have been developed. Such processes conduct heating and compaction of the powder simultaneously, thereby reducing manufacturing time and improving the densification of the component [2,3,4]. Such new sintering processes establish a mechanical-thermal field, which enables a different bonding mechanism between the powder particles [5,6,7]. Hot isostatic pressing (HIP) and field assisted sintering technology (FAST) [8,9,10], are the main names given to such sintering processes. The main difference is on the kind of heating principle [11]. In the HIP sintering, an external heating element, e.g., a heating nozzle, provides the sintering of the pressed compact. In FAST sintering, the sintering heat is provided from the electrical current generating Joule heating while flowing into the compactor and/or the die [12,13]. FAST and HIP operate both the compaction and heating operations inside the closed-die setup. Since the compacted and heated powder cannot expand radially in such a setup, both sintering approaches generate a radial pressure at the die/sample interface. The radial pressure is mainly caused by the axial compaction pressure and thermal heating. The theoretical models, studied by Long and Bockstiegel [14,15], allow an estimation of the radial pressure caused from the axial compaction of the powder. The thermal influence on the radial pressure is studied by applying the thermal expansion law. The radial pressure has a direct impact on the quality of the sintered part because it increases the friction at the interface and, thus, the ejection force. Additionally, the “spring-back” effect may further affect the quality of the sintered component [16]. Several studies have investigated new solutions to reducing the friction, leading to four different types of approaches. A first approach relies on lubricants, which are commonly applied in conventional sintering. The majority of lubricants evaporate above a temperature of 200 °C, leaving, e.g., graphite or molybdenum disulfide as the only options. However, the use of lubricants influences the sample quality in terms of contamination, reducing the “green strength” and density [17,18,19,20]. A second approach is based on the use of a split die solution, where the die includes several dismountable parts [21,22]. The latter are assembled before sintering and then disassembled when the component is sintered and ready to be ejected. A third approach is based on a tapered die solution that makes it possible to gradually decrease the radial pressure, thereby avoiding crack formation [23]. The tapered dies are difficult to manufacture for their complex geometrical shapes. A fourth approach consists of the die pre-stressing, representing a solution being well-known in metal forming. The die pre-stressing reduces the maximum tangential stress, known as the hoop stress, and the stress fluctuations, which the die is subject to. The use of the pre-stress for a sintering tool was justified by the possibility of reducing the internal diameter of the die during the process. Starting from the estimation of the radial pressure, the expected radial expansion of the sample in a free-die wall condition can be predicted [14]. The pre-stress was then designed to achieve an internal diameter reduction being equal to, or larger than, the sample expansion. After sintering, the stress-ring was taken off and the pre-stress was removed from the die which, thus, recovers to the original inner diameter. The radial pressure was decreased, thereby reducing the friction and ejection force. The die pre-stressing can be obtained using more than one approach e.g., stress-rings [24], stress-pins [25], stripwound containers [26], and a SMART^®^ application using shape memory alloys (SMAs) [27,28]. Each of the die pre-stressing solutions features advantages and disadvantages [26,29,30,31].

This paper presents the development of a pre-stressed tool system for the manufacture of micro-gears [32]. The developed pre-stressed tool system worked according to HIP. Fourteen gears were produced using the pre-stressed tool system and then compared to other 14 gears produced without the stress-ring strategy. Different combinations of punch pressure, sintering temperature, and holding time were used to manufacture the gears. The comparison between the two tool systems was based on the ejection force and part fidelity to the computer-aided design (CAD) file. The ejection force was measured during the gear manufacture, while the part fidelity was documented using an optical microscope and computed tomography in order to obtain a multi-scale characterization. Furthermore, porosity analysis was carried out on the gears using computed tomography.

## 2. Materials and Methods

**Figure 1:**
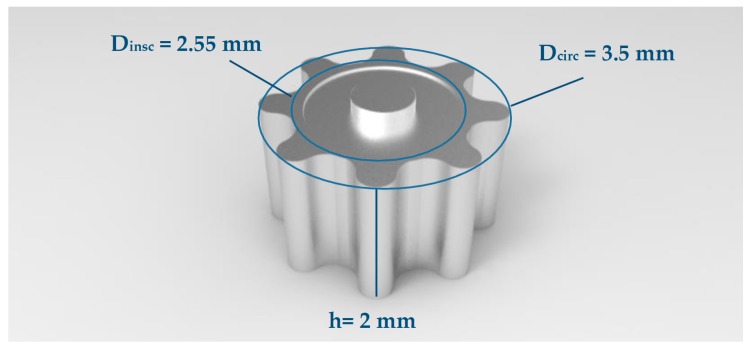
Experimental micro-gear design used in this work.

Figure 1 shows the aluminum micro-gear on which the tooling system was developed in this work. The sintered aluminum powder (AVL Metal Powders, Kortrijk, Belgium) was pure at 99.7%, with a fineness ranging from 42 to 250 µm. The gear had a circumscribed and inscribed circle of 3.5 mm and 2.55 mm, respectively. The height of the gear was of approximately 2 mm. The dimensional tolerances were set in the range of 0.005–0.010 mm. Since 2.7 g/cm^3^ is the assumed aluminum density, the estimated powder weight is 0.038 ± 0.002 g.

**Figure 2:**
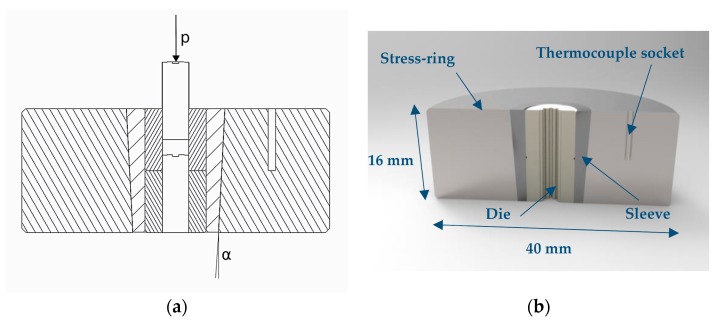
Tool system design for the shrink-fit application: (**a**) stress-ring, sleeve and die section; and (**b**) rendered CAD model of the stress-ring, sleeve and die.

Figure 2 shows the tool system designed for producing the micro gears. The tool system included three elements: stress-ring, sleeve, and die section, see Figure 2a. The die was inserted into the sleeve, and then both were shrink-fitted with the stress-ring, see Figure 2b. The components of the tool were manufactured using electro discharge machining (EDM). The functional surfaces of the three elements of the tool system were all finished to bring the surface roughness down to Ra = 0.2–0.3 µm. The shape at the interference section between the sleeve and the stress-ring was conical to allow the assembling and releasing of the shrink-fit for several sintering cycles. Such a shape approach was based on the experimental investigations of the cold compaction of powder into an elastic die carried out by Noveanu et al. [33]. The tool material was H13 hot-work steel because of its mechanical properties at high temperatures.

The main geometrical characteristics of the designed tool system were the average inner diameter of the stress-ring, *D_stress-ring inn_*, the average outer diameter of the sleeve, *D_sleeve out_*, the resulting interference, *I_Ø_*, the pressure achieved at the sleeve/stress-ring interface, *p_int_*, and the radial reduction obtained at the inner die diameter, *D_red_*. The values of the geometrical characteristics were listed in Table 1.

**Figure 3:**
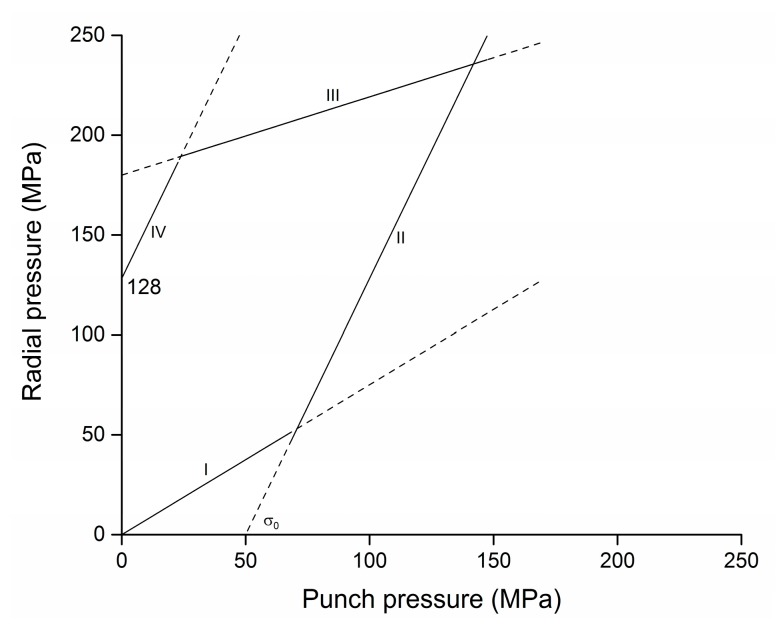
The diagram shows the estimated hysteresis cycle during the axial compaction of the investigated micro-gear according to the Bockstiegel model [15]. The diagram was obtained by assuming an aluminum-steel frictional condition µ = 0.61. Stages: I, elastic loading; II, plastic loading; III, elastic releasing; IV, plastic releasing.

All the geometrical characteristics of the tool system were quantified according to the thick-walled hollow cylinder theory [34] and trade-off choices between the maximum yield strength of the tool material and the estimated total radial pressure arising from the sintering process. The total radial pressure was estimated taking into account the effect of the punch and sintering temperature during the sintering process. The gears were modelled as cylinders having an external diameter as the circumscribed circles. Such a simplification made it possible to design the tool system according to a worst-case pressure scenario. The first component of the radial pressure is the one generated by the punch compaction and was calculated according to Bockstiegel’s model [15], resulting in a hysteresis cycle being shown in Figure 3. By assuming a maximum experimental pressure of 150 MPa for the axial compaction of the powder, the radial pressure at the end of the sintering process was quantified to be 128 MPa.

The second component of radial pressure was originated by the difference in the thermal expansion between the gear and die. The thermal expansion equation was written using a differential way, *dD*, subtracting the two lengths involved for the die and the gear:

(1) $$dD=\left|\left(\alpha_{gear}-\alpha_{die}\right)D_{0}\Delta T\right|$$

Here $\alpha_{gear}\;\mathrm{and}\;\alpha_{die}$ are the thermal expansion coefficients of gear and die, respectively; *D*_0_ is the nominal diameter at the die/gear interface; Δ*T* is the temperature variation with respect to the reference temperature of 20 °C. The maximum experimental sintering temperature was set to be 600 °C. The estimated *dD* corresponded to the theoretical expansion, which would have affected the gear in a wall free-die configuration. *dD*, therefore, represented a deformation in a closed-die. The estimated deformation of the gear generates a radial pressure of 260 MPa according to the Hooke’s elasticity law. Since they were made of the same material, no difference in thermal expansion was assumed among the stress-ring, sleeve, and die.

The total radial pressure was quantified to be 388 MPa by summing the two above mentioned contributions, leading to a theoretical diameter expansion of the gear, *D_exp_*, in a case of wall-free die [14] as follows:

(2) $$D_{exp}=\frac{p_{r}-\nu p_{r}-\nu p_{a}}{E}D_{0}$$

where *p_r_* is the total radial pressure previously calculated; *p_a_* is the axial compaction pressure; *ν* is the Poisson’s ratio; *E* is the Young’s modulus of aluminum. The *D_exp_* value was found to be 13.48 µm. The nearest value to the *D_exp_* value, complying with the yield limit of the used tool material, was chosen as the *D_red_*.

Visual inspections and computed tomography inspections were carried out on the manufactured gears. Visual inspections were carried out to evaluate the impact of the reduction of the ejection force on the surface of the gears using an optical microscope DeMeet-220 (Schut Geometrical Metrology, Groningen, The Netherlands) with a 5:1 lens. The same lighting, fixturing process, and inspection area were used for all the inspected gears. The gears were gently cleaned before being inspected. Computed tomography (CT) inspections were, furthermore, carried out to evaluate the impact of the pre-stress on the resulting porosity and dimensional accuracy of the gears. CT is an imaging method that takes advantage of the capability of X-rays to penetrate the material [35,36].

## 3. Experimental Setup

**Figure 4:**
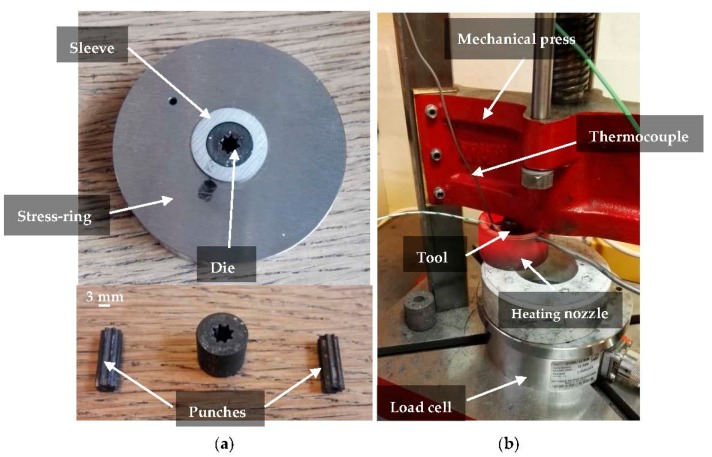
Experimental setup: (**a**) details of the overall pre-stressed tool system, die and punches; and (**b**) detail of the pre-stressed tool configuration during sintering.

Figure 4 shows the developed tool system and its assembly. The tool system was pre-stressed by assembling the die into the sleeve and mounting both into the stress-ring, as shown in Figure 4a. A mechanical press was used to assemble the tools and generate the required fit. The achieved diameter reduction was measured using the optical microscope and found to agree with the estimated value shown in Table 1 in a range of ±2 µm. After assembling, the whole tool was placed into a heating nozzle (Watlov, St. Louis, MO, USA) as shown in Figure 4b. The sintering process was carried out according to the HIP principle. The ejection force representing the parameter of interest in this work was measured using a load cell, U10M (HBM, Darmstadt, Germany).

**Figure 5:**
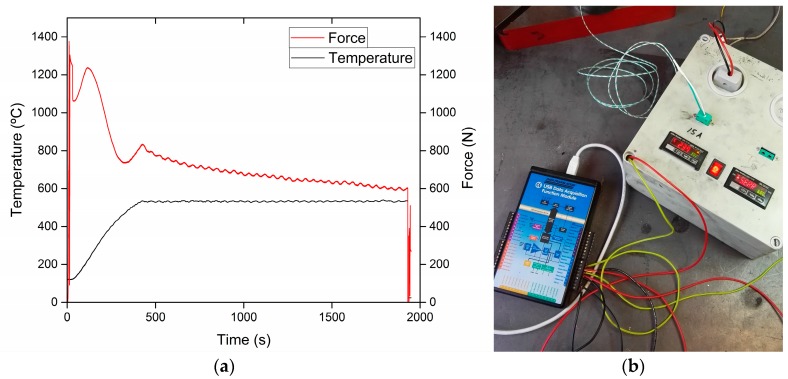
Measuring setup: (**a**) diagram showing the variation of the main process parameters; (**b**) data acquisition system, Data-Translation DT-9800 (Data Translation GmbH, Bietigheim-Bissingen, Germany), and temperature control system, Allen-Bradley 900 TC-32 (Rockwell Automation, Milwaukee, WI, USA).

The designed tool system was tested under different sets of process parameters such as punch pressure (four levels: 75 MPa, 100 MPa, 125 MPa, and 150 MPa), sintering temperature (four levels: 450 °C, 500 °C, 550 °C, and 600 °C), and holding time (four levels: 10 min, 15 min, 20 min, and 25 min). The highest punch pressure and sintering temperature represented the limit conditions to which the designed tool system should be subjected to. The tests were carried out using a partial factorial design, ensuring a randomization of the tests. The punch pressure and sintering temperature were monitored using a data acquisition system (DAQ), Figure 5a. A temperature control system allowed setting the desired sintering temperature for the process, see Figure 5b. The holding time was sampled by using a chronometer. The electronics of the setup was kept at an environment temperature of 21 ± 1 °C, ensuring its stability against thermal drifts. Moreover, the electronics of the setup was rebooted at a step of 25 min to avoid any additional drifts.

Fourteen gears were produced using the pre-stressed tool system and coded as pre-stressed gears (PSGs) throughout the paper. Subsequently, the stress-ring was taken off and the other 14 gears were produced using the same procedures and process parameters used for PSGs. Such gears are coded as no pre-stressed gears (NPSGs) throughout the paper and used as reference conditions. By removing the stress-ring, a non-prestressed tool system with similar geometries and materials to the pre-stressed tool system was realized. The production of the two sets of gears was carried out in both cases using the same sets of sintering process parameters. Molybdenum disulfide (MoS_2_) was used as a lubricant in order to avoid any problem arising from the possible aluminum powder adhering on the tools. Between the manufacture of two gears, the tool system was cooled down, ensuring that the same initial conditions were established for all tests. No effect of the tool wear was considered due to the limited amount of gears manufactured.

The uncertainty of the ejection force measurements was estimated according to the ISO Guide to the Expression of Uncertainty in Measurement (GUM) [37]. The following standard uncertainty contributions: load cell certificate, load cell repeatability when a reference standard was measured, press repeatability, load cell resolution, and eccentric load were considered and modelled using a rectangular distribution [37]. Neither linearity, nor hysteresis, was taken into account because the used measuring range was smaller than 1% of the total measuring range of the used load cell (500 kN). The environment temperature fluctuations were measured, found to be small, compared to other contributions and, thus, neglected. The expanded measurement uncertainty value, with a coverage factor k = 2 at 95% confidence level, was estimated to be in the range of 10–100 N. The largest source of uncertainty was found to be the press repeatability, which was strongly dependent on the process parameters.

## 4. Results and Discussions

**Figure 6:**
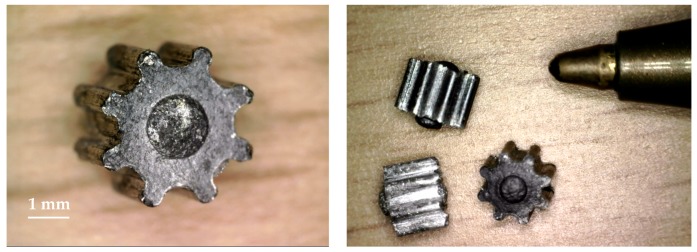
Examples of sintered samples using different process parameters. A pen tip was used to give an idea of the real size of the micro-gears.

**Figure 7:**
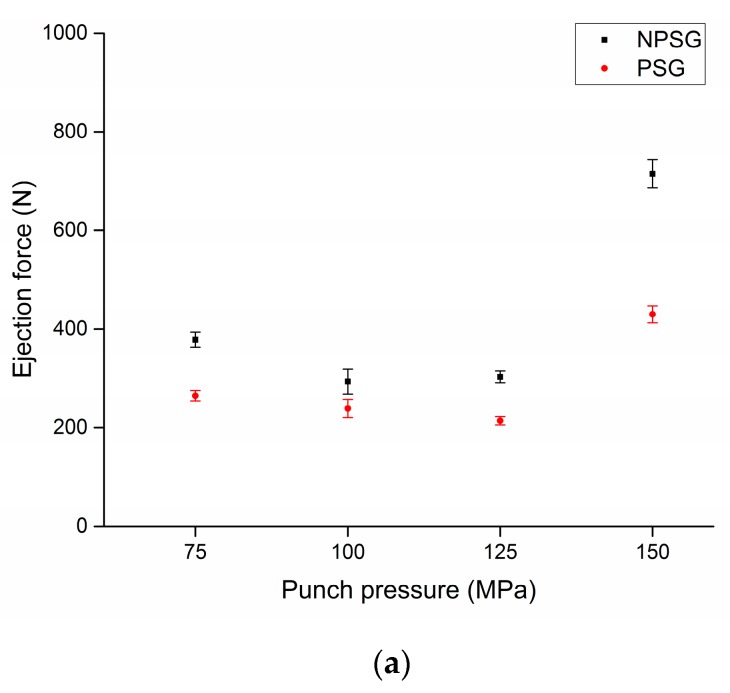

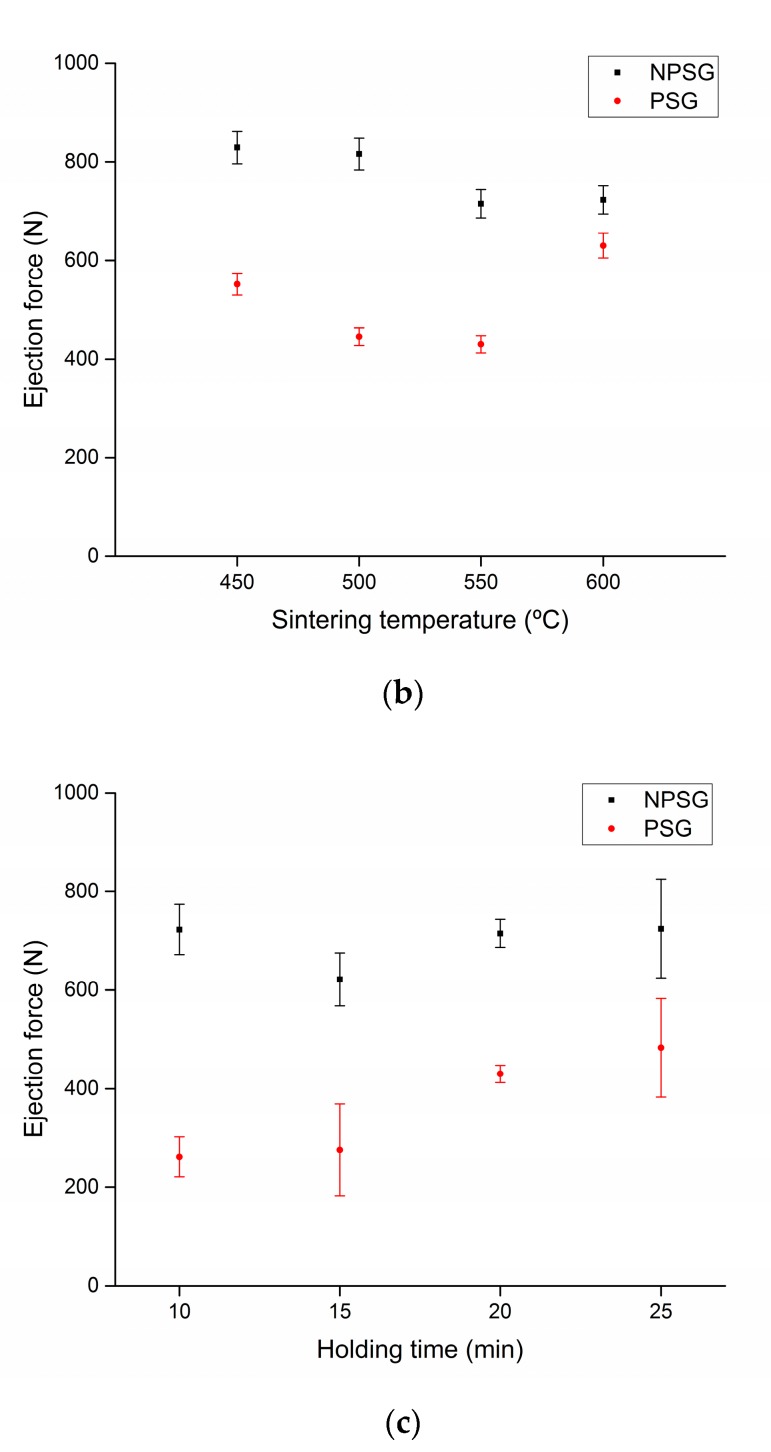
Diagrams showing the experimental values of ejection force measured during the ejection of the micro-gears with respect to the process parameters. (**a**) The four level of punch pressure with constant sintering temperature (550 °C) and holding time (20 min); (**b**) the four level of sintering temperatures with constant punch pressure (150 MPa) and holding time (20 min); and (**c**) the four level of holding time with constant punch pressure (150 MPa) and sintering temperature (550 °C).

Figure 6 shows an example of gears produced using the developed tool system. Figure 7a–c show the evolution of the measured ejection force under different testing conditions. The three series of experimental results were obtained with the punch pressure, sintering temperature and holding time varying once at a time. Each force value was expressed in terms of the average value and expanded measurement uncertainty. By comparing the ejection forces measured between NPSGs and PSGs, it was seen that a strong reduction of the ejection force was achieved for all the considered sintering sensations. The reduction of the ejection force was found to be several times larger than the expanded uncertainty of the ejection force measurements, giving evidence that the reduction had a physical fundament and, thus, can be replicated over the time.

In the case of the PSGs, the lower ejection forces were achieved because of the radial pressure reduction obtained after removing the stress-ring from the tooling system. As a consequence, the die expanded to its designed dimensions, thereby generating a clearance between the tool and gear. Such a clearance resulted in the reduction of the friction and ejection force. The average percentage reduction, *F_red_*, was calculated for each experimental series, i.e., punch pressure, sintering temperature, and holding time, as follows:

(3) $$F_{red}\left[\%\right]=\frac{\sum_{i=1}^{n}\left(\frac{F_{NPSG\;\left(i\right)}-F_{PSG\;\left(i\right)}}{F_{NPSG\;\left(i\right)}}\cdot 100\right)}{n}$$

where *n* is the number of comparisons made for the considered experimental series; *F_NPSG_* and *F_PSG_* are the ejection forces measured for the micro-gears sintered with the same process parameters by using a configuration without and with pre-stress, respectively. A summary of the average results for each experimental series was collected in Table 2. The holding time series showed a reduction of the advantage of the pre-stress as the holding time increased. The longer holding times were, the larger the ejection forces. Such a finding can be due to the fact that longer holding times increased the adhesion of a gear to the die walls, resulting in stronger interactions between the surfaces in contact. A similar percentage force reduction was observed by varying either the punch pressure or the sintering temperature.

### 4.1. Visual Inspection of Micro-Gears Using an Optical Microscope

**Figure 8:**
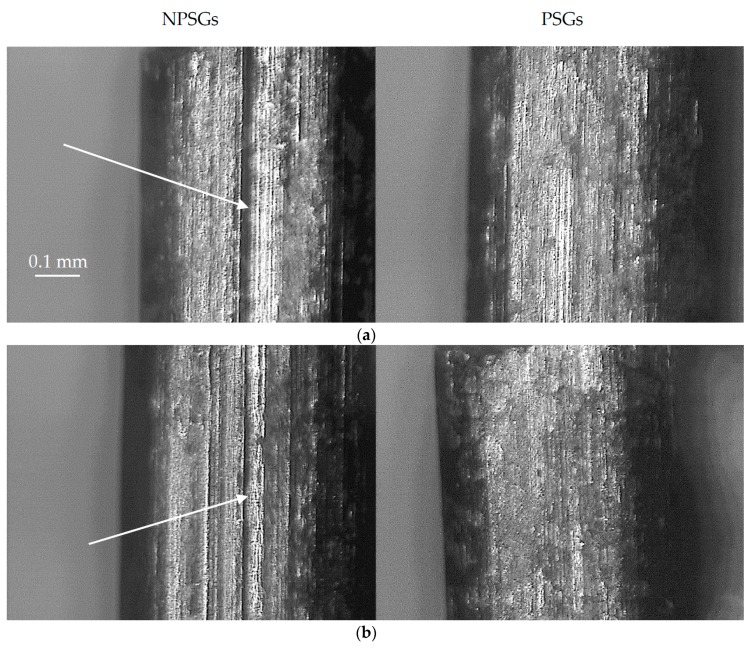

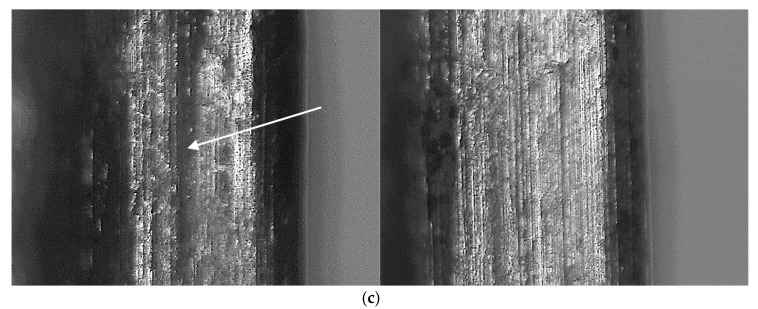
The pictures showing the lateral surfaces of NPSGs, left side, and PSGs, right side, acquired using an optical microscope. PSGs: (**a**) T: 550 °C, holding time: 10 min, punch pressure: 150 MPa, percentage force reduction: −66%; (**b**) T: 550 °C, holding time: 15 min, punch pressure: 150 MPa, percentage force reduction: −64%; and (**c**) T: 550 °C, holding time: 15 min, punch pressure: 150 MPa, percentage force reduction: −30%. Regardless of the sintering process parameters, NPSGs show on their surfaces the consequences of the larger ejections.

Figure 8 shows a collection of pictures of the gears sintered in the present work. PSGs did not show any ploughing effect compared to NPSGs where such effect was well visible, as shown in the figure using arrows. Ploughing was caused by asperities of a tool material metal penetrating into a gear material, which was the softer one. Although it may be deducted that the new developed tool system increased the uniformity of the surface of the gears, the visual inspection did not give sufficient evidences to make a definitive statement. As a consequence, a quantitative analysis was carried out using computed tomography.

### 4.2. Characterization of Micro-Gears Using Computed Tomography

Five repeated scans of a PSG, and five repeated scans of a NPSG were carried out using a Nikon XT H 225 (Nikon, Tokyo, Japan) industrial CT. The two gears were manufactured subsequently in order to avoid any misjudgment due to wear. Table 3 shows an overview of the scanning parameters used in this work. A power of 6 W and a voxel size of 2.8 µm were used for the scanning process, leading to an isotropic scanning resolution of approximately 4 µm. The selected resolution required to minimize the rotary table wobble using a large number of projections and reversal scanner movements designed by the authors. The detector calibration was performed by using a total of 256 projections evenly distributed over four different power levels, with 1024 projections per level. Four power levels were sufficient due to the constant absorption values of the aluminum across the entire spectrum. Due to the high resolution selected, it was necessary to stabilize the used CT for 2 h, otherwise thermal drifts would have impaired the CT measurements. The reconstruction of the projections into a 3D volume was finalized using Nikon Metrology CT PRO 3D version 3.1.9 (Nikon, Tokyo, Japan) without applying any software-based correction features to cope with scanning errors. Surface determination was based on a local thresholding method, implemented in VG studio Max 3.0 (Volume Graphics, Heidelberg, Germany). This method currently represents the state-of-the-art tool for segmenting CT datasets, providing an accuracy of up to 1/10 of the voxel size [38]. The surface was generated by manually selecting the grey values belonging to the gear and to the background on the reconstructed images as seen from different viewpoint angles. A grey value represents the intensity of a voxel [35] and is expressed in terms of shades of grey [39]. Three off-line reference artifacts for systematic errors correction were used to compress the systematic errors and to establish traceability in this work. An off-line reference artefact with a calibrated sphere of 150 µm was used to establish traceability of the porosity and dimensional analysis. Two off-line reference artifacts based on several spheres of 2 mm were used to establish traceability of the dimensional analysis. Since high quality X-ray projections are an essential prerequisite for accurate CT measurements, an image quality analysis was carried out on the X-ray projections. The extent of image noise was quantified and found to be smaller than 0.4% of the average voxel intensity. Furthermore, no missing frequencies were observed in the reconstructed volumes of the gears, guaranteeing that no Feldkamp artifacts [39] compromised the CT measurements. The frequency analysis was performed in the Fourier domain using Fiji post-processing software.

**Figure 9:**
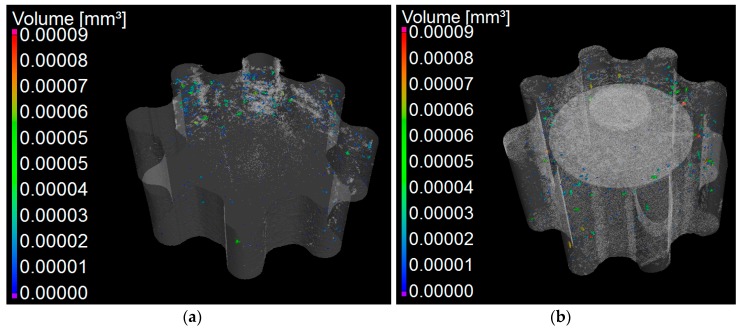
Volume pore distributions for (**a**) NPSG and (**b**) PSG. Deviation range from 0 to 0.00009 mm^3^.

The porosity analysis was performed using two representative 3D volumes, one volume per gear, to minimize the impact of external influences such as dust, image noise, and lubricant. The representative 3D volumes were set to enclose approximately 90% of the total volume of each gear. It was, therefore, assumed that any result obtained from the inspection of the two volumes was representative of the two entire gears. By using the Procedure for Uncertainty MAnagement (PUMA) method [37], the expanded measurement uncertainty, at 95% confidence level, for the porosity analysis was estimated to be equal to ±0.01 mm^3^. The expanded measurement uncertainty was based on the following uncertainty contributions: (i) the uncertainty from the calibration certificates of the off-line reference artefact; (ii) the uncertainty from the measurement repeatability; and (iii) the uncertainty arising from the image noise. All the uncertainty contributions were based on Type B evaluations [37]. The image noise contributed 60% of the expanded measurement uncertainty. Figure 9 shows the spatial porosity distribution for the two gears inspected using CT. The porosity fraction was quantified to be 0.03 ± 0.01% and 0.05 ± 0.01% of the representative volume for PSG and NPSG, respectively. By performing a *t*-test [40], it was found that the two porosity distributions can be assumed to be statistically different, leading to a conclusion that the pre-stress increased the compactness of the powder of which the gears were composed. The *t*-test was conducted at the 95% confidence level and took into account the measurement uncertainty of the porosity fractions. It was also observed that the pores were found to be distributed within the volumes of both selected gears in a different fashion. PSG showed a uniform porosity within the inspected volume, whereas NPSG did not. As a general conclusion, the use of pre-stress reduced the gear porosity by 40% and allowed the achievement of a more uniform spatial distribution of the pores in the gear.

**Figure 10:**
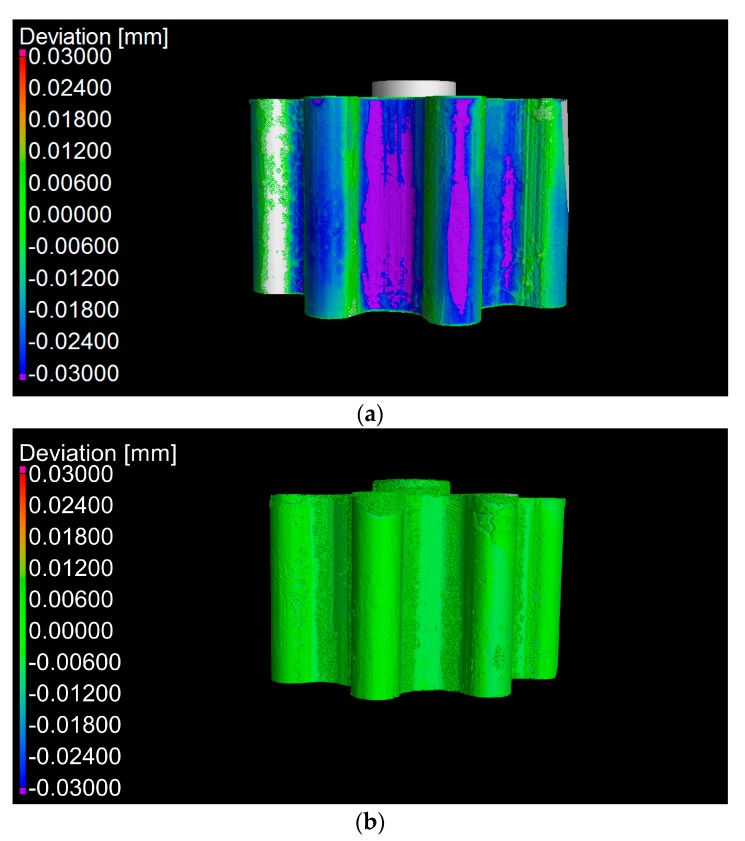
Actual-to-nominal comparison for (**a**) NPSG and (**b**) PSG. Deviation range ±0.03 mm.

The dimensional analysis of the gears was based on the actual-to-nominal comparison tool [40]. The use of the actual-to-nominal comparison allows the quantification of the deviation of each surface point of an object compared to its CAD file [36]. An actual-to-nominal comparison simplifies the measurement procedures compared to other approaches involving primitive features [36]. By using the PUMA method [37], the expanded measurement uncertainty per voxel was calculated, making each point of an actual-to-nominal comparison traceable to the meter. The measurement uncertainty was based on the following uncertainty contributions: (i) the uncertainty from the calibration certificates of the two used off-line reference artifacts; (ii) the uncertainty from the measurement repeatability; (iii) the uncertainty arising from the image noise; and (iv) uncertainty from the post-processing activities. The expanded measurement uncertainty was found to be 0.003 mm for all the voxels of the reconstructed volumes. A single uncertainty value was sufficient in this case due to the following favorable conditions: (i) no gear movements occurred during scanning, establishing that no local reconstruction errors occurred; (ii) the absence of material-related image artifacts, ensuring that X-rays were well modelled during reconstruction at any voxel; (iii) the axial symmetry of the gears, making sure that scatter and the X-ray absorption coefficient did not change locally. The measurement uncertainty per voxel represents a source of novelty, paving the way for 3D measurement uncertainty estimations. Figure 10 shows the two actual-to-nominal comparisons for the two gears with the deviation range and colors. It can be seen from the figures that the two gears appeared to be extremely different, with PSG having the higher fidelity to the CAD file. PSG showed 95% of its surface lied within ±0.009 mm, whereas PSG showed that 95% of its surface lies within ±0.040 mm. By using a *t*-test [40], it was confirmed that the two fidelity values were statistically different. As a general conclusion, the adoption of a pre-stress value can improve the dimensional accuracy of gear by more than 75% compared to gears produced without pre-stress.

## 5. Conclusions

This paper has presented the development of a pre-stressed tool system for the manufacture of micro gears made of aluminum [32]. The developed pre-stressed tool system included a stress-ring and worked according to HIP, thereby reducing the sintering time compared to conventional sintering. Fourteen gears were produced using the pre-stressed tool system and then compared to another 14 gears produced after removing the stress-ring. All the gears were manufactured in an environment kept at of 21 ± 1 °C and using different combinations of punch pressure, sintering temperature, and holding time. By using the newly-developed pre-stressed tool system, a reduction in the ejection force of up to 50% was observed. The magnitude of the ejection force reduction was found to be larger than the ejection force measurement uncertainties, giving evidence that such results had a physical explanation and, thus, can be replicated over time. Visual inspections, carried out using an optical microscope, showed a reduced frictional interaction on the lateral surface of the micro-gears produced by using a pre-stressed tool system. Furthermore, CT measurements documented that the new tool system reduced the porosity in the gear by 40% and improved the dimensional fidelity to CAD by more than 75% compared to gears produced without pre-stress. Finally, the paper has also introduced the concept of CT measurement uncertainty per voxel, as well as proved the applicability of CT to micro-manufacturing with expanded uncertainties of a few microns.

## Figures and Tables

**Table 1:** Dimensional specifications of the pre-stressed tool.

*D_stress-ring inn_*	*D_sleeve out_*	*I_Ø_*	*p_int_*	*D_red_*
11.95 mm	12.00 mm	0.05 mm	326 MPa	12 µm

**Table 2:** Average ejection force reduction during the ejection as a function of the process parameters.

Punch Pressure	Sintering Temperature	Holding Time
−27%	−33%	−50%

**Table 3:** Overview of the scanning parameters used in this work.

Parameter	Unit	Value
X-ray tube voltage	kV	130
X-ray tube current	µA	50
Corrected voxel size	µm	2.8
Magnification factor	-	60
No. of projections	-	2000
No. of image per projection	-	2
Integration time	s	0.5
Scanning time	min	34

## References

[1] Castro R.H.R., Castro R.H.R., van Benthem K. (2013). Overview of Conventional Sintering. Sintering.

[2] Groza J.R., Zavaliangos A. (2003). Nanostructured bulk solids by field activated sintering. Rev. Adv. Mater. Sci..

[3] Wang G.P., Liu W.Q., Huang Y.L., Ma S.C., Zhong Z.C. (2014). Effects of sintering temperature on the mechanical properties of sintered NdFeB permanent magnets prepared by spark plasma sintering. J. Magn. Magn. Mater..

[4] Garay J.E. (2010). Current-Activated, Pressure-Assisted Densification of Materials. Annu. Rev. Mater. Res..

[5] Guillon O., Gonzalez-Julian J., Dargatz B., Kessel T., Schierning G., Rathel J., Herrmann M. (2014). Field-assisted sintering technology/spark plasma sintering: Mechanisms, materials, and technology developments. Adv. Eng. Mater..

[6] Li W., Olevsky E.A., McKittrick J., Maximenko A.L., German R.M. (2012). Densification mechanisms of spark plasma sintering: Multi-step pressure dilatometry. J. Mater. Sci..

[7] Akarachkin S.A., Ivashutenko A.S., Martyushev N.V. (2016). Activation of mass transfer processes at spark plasma sintering of zirconium dioxide. IOP Conf. Ser. Mater. Sci. Eng..

[8] Zhao J., Qin Y., Huang K., Hijji H. (2015). Forming of micro-components by electrical-field activated sintering. MATEC Web Conf..

[9] Orrù R., Licheri R., Locci A.M., Cincotti A., Cao G. (2009). Consolidation/synthesis of materials by electric current activated/assisted sintering. Mater. Sci. Eng. R Rep..

[10] Munir Z.A., Quach D.V., Ohyanagi M. (2011). Electric current activation of sintering: A review of the pulsed electric current sintering process. J. Am. Ceram. Soc..

[11] Delaizir G., Bernard-Granger G., Monnier J., Grodzki R., Kim-Hak O., Szkutnik P.D., Soulier M., Saunier S., Goeuriot D., Rouleau O. (2012). A comparative study of Spark Plasma Sintering (SPS), Hot Isostatic Pressing (HIP) and microwaves sintering techniques on p-type Bi_2_Te_3_ thermoelectric properties. Mater. Res. Bull..

[12] Giuntini D., Olevsky E.A., Garcia-Cardona C., Maximenko A.L., Yurlova M.S., Haines C.D., Martin D.G., Kapoor D. (2013). Localized overheating phenomena and optimization of spark-plasma sintering tooling design. Materials.

[13] Chawake N., Pinto L.D., Srivastav A.K., Akkiraju K., Murty B.S., Kottada R.S. (2014). On Joule heating during spark plasma sintering of metal powders. Scr. Mater..

[14] Long W.M. (1960). Radial pressures in powder compaction. Powder Metall..

[15] Bockstiegel G. The Porosity-Pressure Curve and its Relation to the Size Distribution of Pores in Iron Powder Compacts. Proceedings of the 1965 International Powder Metallurgy Conference.

[16] Höganäs AB Production of Sintered Components. https://www.hoganas.com/globalassets/media/sharepoint-documents/HandbooksAllDocuments/Handbook2_Production_of_Sintered_Components_December_2013_0675HOG_interactive.pdf.

[17] Rahman M.M., Nor S.S.M. (2009). An experimental investigation of metal powder compaction at elevated temperature. Mech. Mater..

[18] Enneti R.K., Lusin A., Kumar S., German R.M., Atre S.V. (2013). Effects of lubricant on green strength, compressibility and ejection of parts in die compaction process. Powder Technol..

[19] Li Y.Y., Ngai T.L., Zhang D.T., Long Y., Xia W. (2002). Effect of die wall lubrication on warm compaction powder metallurgy. J. Mater. Process. Technol..

[20] Luo S.D., Yang Y.F., Schaffer G.B., Qian M. (2014). Warm die compaction and sintering of titanium and titanium alloy powders. J. Mater. Process. Technol..

[21] Chen P., Kim G.-Y., Ni J. (2007). Investigations in the compaction and sintering of large ceramic parts. J. Mater. Process. Technol..

[22] Andresen H., Lund E. Tooling Solutions for Cold and Warm Forging Applications for Automotive and Other Segments. http://www.istma.org/istma-world/ISTMA_Conferencehall/uddeholm2008/Tooling%20solutions%20for%20cold%20and%20warm%20forging_Erik%20Lund_Henrik%20Andresen.pdf.

[23] Garner S., Ruiz E., Strong J., Zavaliangos A. (2014). Mechanisms of crack formation in die compacted powders during unloading and ejection: An experimental and modeling comparison between standard straight and tapered dies. Powder Technol..

[24] Armentani E., Bocchini G.F., Cricrì G. (2012). Doubly shrink fitted dies: Optimisation by analytical and FEM calculations. Powder Metall..

[25] Koç M., Arslan M.A. (2003). Design and finite element analysis of innovative tooling elements (stress pins) to prolong die life and improve dimensional tolerances in precision forming processes. J. Mater. Process. Technol..

[26] Groenbaek J., Nielsen E.R. (1997). Stripwound containers for combined radial and axial prestressing. J. Mater. Process. Technol..

[27] Pan W., Qin Y., Law F., Ma Y., Brockett A., Juster N. (2008). Feasibility study and tool design of using shape memory alloy as tool-structural elements for forming-error compensation in microforming. Int. J. Adv. Manuf. Technol..

[28] Qin Y. (2006). Forming-tool design innovation and intelligent tool-structure/system concepts. Int. J. Mach. Tools Manuf..

[29] Fu M.W., Chan W.L., Fu M.W., Chan W.L. (2014). Micro-Scaled Products Development via Microforming.

[30] Ghassemali E., Tan M.J., Jarfors A.E.W., Lim S.C.V. (2013). Progressive microforming process: Towards the mass production of micro-parts using sheet metal. Int. J. Adv. Manuf. Technol..

[31] Johnson D., Martynov V., Gupta V. (2001). Applications of shape memory alloys: Advantages, disadvantages, and limitations. Proc. SPIE.

[32] Cannella E., Nielsen E.K., Arentoft M. Ejection force analysis of sintered aluminium micro gears using a shrink-fit die principle. Proceedings of the 11th International Conference on Multi-Material Micro Manufacture (4M2016): Co-organised with 10th International Workshop on Microfactories (IWMF2016).

[33] Noveanu D. (2013). Researches concerning a new method for obtaining spur gears by metal powder compaction in elastic dies. Metalurgia.

[34] Atzori B. (2001). Gusci spessi. Appunti di Costruzione di Macchine.

[35] Kruth J.P., Bartscher M., Carmignato S., Schmitt R., De Chiffre L., Weckenmann A. (2011). Computed tomography for dimensional metrology. CIRP Ann. Manuf. Technol..

[36] De Chiffre L., Carmignato S., Kruth J.P., Schmitt R., Weckenmann A. (2014). Industrial applications of computed tomography. CIRP Ann. Manuf. Technol..

[37] (2011). Geometrical Product Specifications (GPS)—Inspection by Measurement of Workpieces and Measuring Equipment—Part 2: Guidance for the Estimation of Uncertainty in GPS Measurement, in Calibration of Measuring Equipment and in Product.

[38] Müller P. (2013). Coordinate Metrology by Traceable Computed Tomography.

[39] Stolfi A. (2017). Integrated Quality Control of Precision Assemblies Using Computed Tomography. Ph.D. Thesis.

[40] Witt P.L., McGrain P. (1985). Comparing two sample means t tests. Phys. Ther..

[41] MICRO-FAST. www.micro-fast.eu.

